# Supplementary material for: Rethinking procedural pain in labor: A comparison of lidocaine injection techniques for epidural catheter placement assessed with an objective clinician-centric pain score—A double-blind randomized controlled trial
Source: PLoS One. 2026 Feb 11;21(2):e0339664. doi: 10.1371/journal.pone.0339664 (PMC12893574; doi:10.1371/journal.pone.0339664)
Supplement: S1 Protocol — (PDF) [file pone.0339664.s002.pdf]

From: Reeves, Jeremy
To: Reeves, Jeremy
Subject: FW: Initial Submission Approved for #2023H0250
Date: Monday, August 28, 2023 10:41:43 AM

From: OR IRB Info <IRBInfo@osu.edu>
Sent: Monday, August 28, 2023 10:40:32 AM (UTC-05:00) Eastern Time (US & Canada)
To: Hu, Ling <hu.1945@osu.edu>
Cc: Uribe, Alberto <uribe.9@osu.edu>; Reeves, Jeremy <reeves.331@osu.edu>; Vera Miquilena, Elvia <veramiquilena.1@osu.edu>
Subject: Initial Submission Approved for #2023H0250

The Ohio State University

Biomedical Sciences
Institutional Review Board

130C Mount Hall
1050 Carmack Road
Columbus, OH 43210-1002

orrp.osu.edu

08/28/2023

Study Number: 2023H0250
Study Title: The Evaluation of Pain Associated to The Epidural Tuohy Needle Insertion with Prior Administration of Subcutaneous or Intradermal Lidocaine in Parturient Women Requesting Epidural Analgesia for Laboring Pain.

Type of Review: Initial Submission

Review Method: Convened

Date of IRB Approval: 08/26/2023
Date of IRB Approval Expiration: 08/26/2024

Dear Ling Hu,

The Ohio State Biomedical Sciences IRB **APPROVED** the above referenced research.

In addition, the following were also approved for this study:

- Pregnant Women/Fetuses
- Partial Waiver of HIPAA Research Authorization

Administrative note: The Biomedical IRB determined that this research presents no more than minimal risk to participants. When submitting applications for continuing review, please request expedited review.

As Principal Investigator, you are responsible for ensuring that all individuals assisting in the conduct of the study are informed of their obligations for following the IRB-approved protocol

and applicable regulations, laws, and policies, including the obligation to report any problems or potential noncompliance with the requirements or determinations of the IRB. Changes to the research (e.g., recruitment procedures, advertisements, enrollment numbers, etc.) or informed consent process must be approved by the IRB before implemented, except where necessary to eliminate apparent immediate hazards to subjects.

This approval is issued under The Ohio State University's OHRP Federalwide Assurance #00006378 and is valid until the expiration date listed above. ***Without further review, IRB approval will no longer be in effect on the expiration date.*** To continue the study, a continuing review application must be approved before the expiration date to avoid a lapse in IRB approval and the need to stop all research activities. A final study report must be provided to the IRB once all research activities involving human subjects have ended.

Records relating to the research (including signed consent forms) must be retained and available for audit for at least 5 years after the study is closed. For more information, see university policies, Institutional Data and Research Data.

Human research protection program policies, procedures, and guidance can be found on the ORRP website.

Karla Zadnik, OD, PhD

Karla Zadnik, OD, PhD, Chair
Ohio State Biomedical Sciences IRB

AAHRPP
