# Supplementary material for: Rethinking procedural pain in labor: A comparison of lidocaine injection techniques for epidural catheter placement assessed with an objective clinician-centric pain score—A double-blind randomized controlled trial
Source: PLoS One. 2026 Feb 11;21(2):e0339664. doi: 10.1371/journal.pone.0339664 (PMC12893574; doi:10.1371/journal.pone.0339664)
Supplement: Attachment — Submitted filename: Response to Reviewers.docx [file pone.0339664.s004.docx]

# Response to Reviewers

1. **Comment:** Please ensure that your manuscript meets PLOS ONE's style requirements, including those for file naming. The PLOS ONE style templates can be found at [https://journals.plos.org/plosone/s/file?id=wjVg/PLOSOne_formatting_sample_main_body.pdf](https://nam12.safelinks.protection.outlook.com/?url=https%3A%2F%2Furldefense.com%2Fv3%2F__https%3A%2F%2Fjournals.plos.org%2Fplosone%2Fs%2Ffile%3Fid%3DwjVg*PLOSOne_formatting_sample_main_body.pdf__%3BLw!!AU3bcTlGKuA!BI7ceTyo_U6TIIzr7MDKcYF8hzihn3_h3bgTkNrOhk2ZuVtTzUdHeNjerzfogYbTNCBUPgWYqgH6evqGfYXz%24&data=05%7C02%7CLukas.Croner%40osumc.edu%7Cc46b07b961284011b9b908de1b9f0df6%7C0b95a125791c4f0a9f9e99e363117506%7C0%7C0%7C638978567116427577%7CUnknown%7CTWFpbGZsb3d8eyJFbXB0eU1hcGkiOnRydWUsIlYiOiIwLjAuMDAwMCIsIlAiOiJXaW4zMiIsIkFOIjoiTWFpbCIsIldUIjoyfQ%3D%3D%7C0%7C%7C%7C&sdata=ApsR45mtQ6fwo%2BygBUeA4jKs1t8RvBhkVJoO3wugFpU%3D&reserved=0) and [https://journals.plos.org/plosone/s/file?id=ba62/PLOSOne_formatting_sample_title_authors_affiliations.pdf](https://nam12.safelinks.protection.outlook.com/?url=https%3A%2F%2Furldefense.com%2Fv3%2F__https%3A%2F%2Fjournals.plos.org%2Fplosone%2Fs%2Ffile%3Fid%3Dba62*PLOSOne_formatting_sample_title_authors_affiliations.pdf__%3BLw!!AU3bcTlGKuA!BI7ceTyo_U6TIIzr7MDKcYF8hzihn3_h3bgTkNrOhk2ZuVtTzUdHeNjerzfogYbTNCBUPgWYqgH6erboAEyy%24&data=05%7C02%7CLukas.Croner%40osumc.edu%7Cc46b07b961284011b9b908de1b9f0df6%7C0b95a125791c4f0a9f9e99e363117506%7C0%7C0%7C638978567116463367%7CUnknown%7CTWFpbGZsb3d8eyJFbXB0eU1hcGkiOnRydWUsIlYiOiIwLjAuMDAwMCIsIlAiOiJXaW4zMiIsIkFOIjoiTWFpbCIsIldUIjoyfQ%3D%3D%7C0%7C%7C%7C&sdata=ZNDNlMDAQ8IE18rm1DYBduuqOyBlTLNraHXX%2B7PMAtM%3D&reserved=0) -

**Response:** We have revised the manuscript to comply with PLOS ONE’s formatting guidelines for both the main text and title/author sections.

2. **Comment:** In the online submission form, you indicated that the de-identified dataset generated and analyzed during the current study is available from the corresponding author upon reasonable request. The study protocol and statistical code used for analysis are also available upon request.

All PLOS journals now require all data underlying the findings described in their manuscript to be freely available to other researchers, either 1. In a public repository, 2. Within the manuscript itself, or 3. Uploaded as supplementary information.
This policy applies to all data except where public deposition would breach compliance with the protocol approved by your research ethics board. If your data cannot be made publicly available for ethical or legal reasons (e.g., public availability would compromise patient privacy), please explain your reasons on resubmission and your exemption request will be escalated for approval.

**Response:** The de-identified raw dataset has been uploaded as Supporting Information in accordance with PLOS ONE’s data-sharing policy. All personal identifiers have been removed to ensure participant privacy.

3. **Comment:** We note that the grant information you provided in the ‘Funding Information’ and ‘Financial Disclosure’ sections do not match.

When you resubmit, please ensure that you provide the correct grant numbers for the awards you received for your study in the ‘Funding Information’ section.

**Response:** The funding details have been corrected and now match across the “Funding Information” and “Financial Disclosure” sections.

4. **Comment:** Please include a caption for figure 1.

**Response:** A descriptive caption for Figure 1 has been added and the figure re-uploaded: “The above figure shows the randomization of 51 patients into two interventions:

Intradermal Lidocaine (25 participants) and Subcutaneous Lidocaine (26 participants).

All participants were analyzed according to original group allocation.”

5. **Comment:** [Please include captions for your Supporting Information files at the end of your manuscript, and update any in-text citations to match accordingly. Please see our Supporting Information guidelines for more information: http://journals.plos.org/plosone/s/supporting-information.](https://nam12.safelinks.protection.outlook.com/?url=http%3A%2F%2Ftrack.editorialmanager.com%2FCL0%2Fhttp%3A%252F%252Fjournals.plos.org%252Fplosone%252Fs%252Fsupporting-information.%2F1%2F010f019a4d009732-0b2cb152-ca63-473d-af25-2d714f05ff5b-000000%2FTg7hJFXYuBRvaDduUnvvMDbTBTzbGF5-JmkfgIeYHhE%3D235&data=05%7C02%7CLukas.Croner%40osumc.edu%7Cc46b07b961284011b9b908de1b9f0df6%7C0b95a125791c4f0a9f9e99e363117506%7C0%7C0%7C638978567116486671%7CUnknown%7CTWFpbGZsb3d8eyJFbXB0eU1hcGkiOnRydWUsIlYiOiIwLjAuMDAwMCIsIlAiOiJXaW4zMiIsIkFOIjoiTWFpbCIsIldUIjoyfQ%3D%3D%7C0%7C%7C%7C&sdata=tQlK9FyAVBm30Xw15NBW0ek5gQBT%2FPTZoO1mgmQi%2FMw%3D&reserved=0)

**Response:** Titles and captions for all Supporting Information files have been included at the end of the manuscript, and in-text citations have been updated accordingly.

6. **Comment:** We note that there is identifying data in the Supporting Information file <Initial Submission Approved. Pdf, Initial Submission Approved Protocol. pdf>. Due to the inclusion of these potentially identifying data, we have removed this file from your file inventory. Prior to sharing human research participant data, authors should consult with an ethics committee to ensure data are shared in accordance with participant consent and all applicable local laws.

Data sharing should never compromise participant privacy. It is therefore not appropriate to publicly share personally identifiable data on human research participants. The following are examples of data that should not be shared:

-Name, initials, physical address
-Ages more specific than whole numbers
-Internet protocol (IP) address
-Specific dates (birth dates, death dates, examination dates, etc.)
-Contact information such as phone number or email address
-Location data
-ID numbers that seem specific (long numbers, include initials, titled “Hospital ID”) rather than random (small numbers in numerical order)

Data that are not directly identifying may also be inappropriate to share, as in combination they can become identifying. For example, data collected from a small group of participants, vulnerable populations, or private groups should not be shared if they involve indirect identifiers (such as sex, ethnicity, location, etc.) that may risk the identification of study participants.

Additional guidance on preparing raw data for publication can be found in our Data Policy ([https://journals.plos.org/plosone/s/data-availability#loc-human-research-participant-data-and-other-sensitive-data](https://nam12.safelinks.protection.outlook.com/?url=https%3A%2F%2Furldefense.com%2Fv3%2F__https%3A%2F%2Fjournals.plos.org%2Fplosone%2Fs%2Fdata-availability*loc-human-research-participant-data-and-other-sensitive-data__%3BIw!!AU3bcTlGKuA!BI7ceTyo_U6TIIzr7MDKcYF8hzihn3_h3bgTkNrOhk2ZuVtTzUdHeNjerzfogYbTNCBUPgWYqgH6eqg0khmz%24&data=05%7C02%7CLukas.Croner%40osumc.edu%7Cc46b07b961284011b9b908de1b9f0df6%7C0b95a125791c4f0a9f9e99e363117506%7C0%7C0%7C638978567116506717%7CUnknown%7CTWFpbGZsb3d8eyJFbXB0eU1hcGkiOnRydWUsIlYiOiIwLjAuMDAwMCIsIlAiOiJXaW4zMiIsIkFOIjoiTWFpbCIsIldUIjoyfQ%3D%3D%7C0%7C%7C%7C&sdata=mJ%2FXVlUTO1xuYGHLzM5jm3FNLlkoky%2BW%2FLhjfg%2FXvEI%3D&reserved=0)) and in the following article: [http://www.bmj.com/content/340/bmj.c181.long](https://nam12.safelinks.protection.outlook.com/?url=https%3A%2F%2Furldefense.com%2Fv3%2F__http%3A%2F%2Fwww.bmj.com%2Fcontent%2F340%2Fbmj.c181.long__%3B!!AU3bcTlGKuA!BI7ceTyo_U6TIIzr7MDKcYF8hzihn3_h3bgTkNrOhk2ZuVtTzUdHeNjerzfogYbTNCBUPgWYqgH6eu5WKnhx%24&data=05%7C02%7CLukas.Croner%40osumc.edu%7Cc46b07b961284011b9b908de1b9f0df6%7C0b95a125791c4f0a9f9e99e363117506%7C0%7C0%7C638978567116529187%7CUnknown%7CTWFpbGZsb3d8eyJFbXB0eU1hcGkiOnRydWUsIlYiOiIwLjAuMDAwMCIsIlAiOiJXaW4zMiIsIkFOIjoiTWFpbCIsIldUIjoyfQ%3D%3D%7C0%7C%7C%7C&sdata=tqW7TxgrzOEpasx7jeZ8LyVb64foms9GjaVJvwpAizI%3D&reserved=0).

Please remove or anonymize all personal information (<specific identifying information in file to be removed>), ensure that the data shared are in accordance with participant consent, and re-upload a fully anonymized data set. Please note that spreadsheet columns with personal information must be removed and not hidden as all hidden columns will appear in the published file.

**Response:** All identifying information has been removed from the Supporting Information files. The uploaded dataset is fully anonymized and complies with participant consent and ethical standards.

7. **Comment:** If the reviewer comments include a recommendation to cite specific previously published works, please review and evaluate these publications to determine whether they are relevant and should be cited. There is no requirement to cite these works unless the editor has indicated otherwise.

**Response:** We have addressed all reviewer suggestions:

- Clarified limitations, emphasizing the small sample size and single-center design.
  - Added statements in the limitations section conclusion to caution interpretation of significant findings and highlight the need for larger studies: “Third, the CPOT was originally designed for use in critical care settings and has not been validated for assessing pain during labor and delivery procedures; its reliability in this context remains to be established, particularly comparisons of individual components, such as muscle tension alone” and “However, given small sample size, more research is necessary to validate these findings.”
- Clarified “repeat” measures for linear mixed effect models: “For hemodynamics, linear mixed-effects models were used to account for measurements at three time points per participant: baseline, after lidocaine injection, and after Tuohy needle insertion”
  - Added clarifying statement to results: “Pairwise comparisons of hemodynamics were made between intradermal and subcutaneous injection techniques at three timepoints: baseline, after lidocaine injection, and after Tuohy insertion.”
  - Also updated p-values in Table 4 for precision.
